# Glucose and lipid metabolism in non-diabetic, non-obese patients with obstructive sleep apnea: sex differences

**DOI:** 10.3389/fnut.2025.1619371

**Published:** 2025-08-20

**Authors:** Yuhan Wang, Beini Zhou, Wuriliga Yue, Mengcan Wang, Ke Hu

**Affiliations:** Department of Respiratory and Critical Care Medicine, Renmin Hospital of Wuhan University, Wuhan, China

**Keywords:** obstructive sleep apnea, glucose, lipid, triglyceride-glucose index, diabetes, obesity, sex

## Abstract

**Objective:**

Obstructive sleep apnea (OSA) is associated with glucose and lipid disturbances and insulin resistance. However, glucose and lipid disturbances and insulin resistance in OSA are often attributed to confounding obesity and/or diabetes. Studies on nondiabetic, nonobese OSA patients are very limited.

**Methods:**

This cross-sectional study retrospectively analyzed non-diabetic, non-obese adults who underwent a home sleep apnea testing and collected fasting blood samples before or after the sleep study to measure glucose and lipids. This study was designed as a cross-sectional study and therefore can only demonstrate associations between variables, but not causality.

**Results:**

Among the 191 participants (mean age 48.94 years, 68.06% male) included in the study, 83.77% had OSA. The high-density lipoprotein cholesterol (HDL-C) level in OSA participants was significantly lower (0.99 vs. 1.12 mmol/L, *p* = 0.036), and the triglyceride-glucose (TyG) index was significantly higher (8.74 vs. 8.45, *p* = 0.016), while there was no significant difference in the levels of total cholesterol, low-density lipoprotein cholesterol (LDL-C), and non-HDL-C. Correlation analysis by sex showed that AHI was significantly positively correlated with fasting plasma glucose (*r* = 0.373), non-HDL-C (*r* = 0.280), and TyG index (*r* = 0.337) in female participants, while AHI was only significantly negatively correlated with HDL-C (*r* = −0.194) in male participants. Multivariable analysis revealed that compared with non-OSA individuals, OSA severity in women was independently associated with fasting plasma glucose (AHI ≥ 5: *β* = 0.55, 95 % CI 0.13 to 0.98; AHI ≥ 15: *β* = 0.60, 95% CI 0.13 to 1.07) and TyG index (AHI ≥ 5: *β* = 0.37, 95% CI 0.08 to 0.66; AHI ≥ 15: *β* = 0.39, 95% CI 0.07 to 0.71; AHI ≥ 30: *β* = 0.53, 95% CI 0.08 to 0.98). In contrast, among men, OSA severity showed independent associations with triglycerides (15 ≤ AHI < 30: *β* = 1.00, 95% CI 0.05 to 1.95) and HDL-C (AHI ≥ 15: *β* = −0.17, 95% CI -0.33 to −0.01; AHI ≥ 30: *β* = −0.22, 95% CI -0.38 to −0.06).

**Conclusion:**

Our study supports the claim that there are sex differences in glucose and lipid metabolic disorders in non-diabetic, non-obese OSA participants: women mainly showed elevated fasting plasma glucose and TyG index, while men showed dyslipidemia with elevated triglycerides and decreased HDL-C. These findings highlight the need to consider sex differences when assessing OSA-related metabolic risks.

## 1 Introduction

Obstructive sleep apnea (OSA) is the most common sleep-disordered breathing with a high prevalence worldwide, especially in obese people (1). Compared with normal controls with similar body mass index (BMI), patients with acromegaly have a significantly increased risk of OSA, which is mainly attributed to their craniofacial deformities, macroglossia, and thickening of the pharyngeal wall caused by soft tissue hyperplasia (2). This confirms that abnormal upper airway anatomy increases the risk of pharyngeal collapse. Despite the presence of severe OSA, most Chinese patients are not obese, and their craniofacial features are associated with an increased risk of OSA (3). The main characteristics of OSA are intermittent hypoxia, sleep fragmentation, and oxidative stress during sleep (4). OSA has been shown to be associated with the morbidity and mortality of cardiovascular and metabolic diseases, and metabolic dysfunction may be an important driving factor for cardiovascular disease in OSA participants (5, 6).

Several clinical observational studies have revealed the association between OSA and dyslipidemia. Data from the European Sleep Apnea Database showed that OSA severity was independently associated with cholesterol and triglyceride concentrations (7). Large-scale observational studies from China further found that there was a nonlinear, multi-stage dose-effect relationship between OSA and dyslipidemia, and quantified the strength of the association between different OSA severity stages and specific lipid risk factors (8). In addition, differences in the use of lipid-lowering drugs, dietary habits, and genetic background may also lead to differences in the prevalence of hypercholesterolemia in different regions, which has been confirmed in the regional differences in lipid status in different parts of Europe (7).

Dyslipidemia is particularly common in obese people, and most obese patients have lipid metabolism disorders (9). At the same time, type 2 diabetes mellitus (T2DM) is also associated with dyslipidemia, including decreased high-density lipoprotein cholesterol (HDL-C) levels and increased low-density lipoprotein cholesterol (LDL-C) and triglyceride levels (9). Obesity and diabetes are risk factors for OSA, and their relationship with OSA may be bidirectional (10, 11). Not surprisingly, obesity and diabetes, as important confounders, play an important role in the development of dyslipidemia in OSA patients. However, most previous studies did not exclude patients with diabetes and obesity, a major confounder that may bias the research results. Secondly, obese patients are more likely to seek clinical attention, while Chinese patients are less likely to be obese and more likely to have craniofacial variations that predispose to OSA, which further leads to insufficient data representation of non-obese OSA individuals in clinical cohorts (12).

To date, there is a lack of research on Chinese non-diabetic and non-obese OSA patients. It is worth noting that although the severity of OSA in women is generally milder, they are more likely to have adverse clinical outcomes compared with men (13). Therefore, this study further analyzed the effects of sex on glucose and lipid metabolism in patients with OSA. We hypothesized that there would be significant sex differences in the metabolic characteristics of OSA patients.

## 2 Methods

### 2.1 Study design and study population

The participants of this study were suspected OSA participants who received home sleep apnea testing (HSAT) at the Respiratory and Sleep Medicine Center, Renmin Hospital of Wuhan University from January to June 2024. Inclusion criteria: patients aged 18 years and above, hospitalized for respiratory, cardiovascular and other diseases. During the stable stage of the disease, patients underwent sleep monitoring due to complaints of snoring, daytime sleepiness or unexplained hypertension. Exclusion criteria: (1) participants receiving continuous positive airway pressure (CPAP); (2) participants using psychotropic drugs that may affect sleep (including antidepressants, sedatives, or hypnotics); (3) obese participants with a BMI ≥ 30 kg/m^2^; and (4) participants with T2DM, including fasting plasma glucose (FPG) ≥ 7.0 mmol/L or a history of confirmed diagnosis of T2DM or a history of hypoglycemic drug treatment. Data for this retrospective study were collected retrospectively from medical records at specific time points. Due to the inherent limitations of the retrospective study design, some data were incomplete, so cases with missing values were excluded from the final analysis. Finally, 191 consecutive non-diabetic, non-obese hospitalized participants with suspected OSA were included for analysis.

This study adhered to the Declaration of Helsinki. The design of this retrospective study was approved by the Ethics Committee of Renmin Hospital of Wuhan University. Due to the retrospective nature of the study, the requirement for informed consent was waived and the identity of the participants remained anonymous.

### 2.2 Sleep studies

The study used the OrbSense sleep apnea monitor (Megasens Technology, Hangzhou, China) for sleep studies. The device consists of a bioradar sensor and a pulse oximeter. Our team has used this device in many studies (14–16). The device was placed at the patient’s bedside (≤100 cm away and ≥10 cm high) and recorded ≥3 h of sleep duration. The device analyzed respiratory events using a proprietary algorithm to obtain key parameters such as apnea-hypopnea index (AHI), mean oxygen saturation by pulse oximetry (mean SpO_2_), lowest oxygen saturation by pulse oximetry (LSpO_2_), oxygen desaturation index (ODI, defined as the number of times per hour that oxygen levels dropped ≥ 4% from baseline), the percentage of total sleep time (TST) with oxygen saturation < 90% (T90, %). Professionals will generate a report the day after the sleep monitoring. The device is comparable to polysomnography in detecting OSA (17, 18). This study used the 2012 version of the American Academy of Sleep Medicine (AASM) criteria to diagnose OSA, with AHI ≥ 5/h as the diagnostic criterion, and the disease was divided into mild (5– < 15/h), moderate (15– < 30/h), and severe (≥30/h) according to the AHI (19).

### 2.3 Fasting plasma glucose, lipid profiles, and insulin resistance

Fasting blood samples collected before and after the sleep study to measure glucose and lipids were retrospectively analyzed. These blood samples included FPG, serum total cholesterol, triglycerides, HDL-C, and LDL-C, were retrospectively collected from the electronic medical record system. Non-HDL-C was calculated as the difference between total cholesterol and HDL-C levels. The TyG index was calculated as ln [Triglycerides (mg/dL) × FPG (mg/dL) /2]. Due to the retrospective design of this study, fasting insulin data were not available, so the homeostasis model assessment of insulin resistance (HOMA-IR) index could not be calculated. However, the TyG index has been shown to be a surrogate marker for identifying insulin resistance in healthy participants (20, 21).

### 2.4 Covariates

Smoking history, alcohol drinking, hypertension, cardiovascular disease, stroke, and the use of lipid-lowering drugs were retrospectively collected from the electronic medical record system.

### 2.5 Statistical analysis

Categorical variables were expressed as numbers (percentage, %), and continuous variables were described as means (standard deviation, SD) or medians (interquartile range, IQR), as appropriate. One-way analysis of variance (normal distribution), Kruskal-Wallis test (skewed distribution), and chi-square test (categorical variables) were used to compare differences between groups. The severity of OSA was measured according to AHI. Because AHI and glucose, lipids, and TyG index were not all normally distributed, Spearman correlation analysis was used to determine the relationship between these parameters.

We further used nonparametric statistical methods to analyze the differences in glucose, lipid profiles, and TyG index between different OSA severity groups in males and females. First, the Kruskal-Wallis test was used for overall comparison between groups. When the test results were statistically significant, Dunn’s *post hoc* test was further used for pairwise comparisons. The Benjamini-Hochberg method was used to correct the *p*-values for multiple tests, and the corrected significance levels were marked with asterisks between the significant difference groups. In addition, multivariable regression analysis was performed to determine the relationship between OSA severity and glucose and lipid metabolism, and stratified analysis was performed according to sex. All models were adjusted for age, sex, BMI, smoking history, alcohol drinking, hypertension, cardiovascular disease, stroke, and use of lipid-lowering drugs, in addition to the stratification factors themselves.

All analyses were performed using the statistical software packages R (The R Foundation)^1^ and EmpowerStats (X&Y Solutions, Inc., Boston, MA).^2^ *p* value of < 0.05 was considered significant by two-tailed tests.

## 3 Results

Table 1 compares the baseline characteristics of OSA participants (*n* = 160, 83.77%) and non-OSA participants (*n* = 31, 16.23%). The results showed that there were no significant differences between the two groups in terms of age, FPG, total cholesterol, triglycerides, non-HDL-C, and LDL-C (all *p* > 0.05). However, the OSA group had a higher BMI (25.41 vs. 24.27 kg/m^2^, *p* = 0.056, borderline significant) and higher triglycerides (1. 56 vs. 1. 23 mmol/L, *p* = 0.055, borderline significant), and the AHI and ODI were more severe, the mean SPO₂ and LSPO₂ were lower, and the proportion of T90 was greater (all *p* < 0.01). In terms of lipid metabolism, the HDL-C of the OSA group was significantly lower (0.99 vs. 1.12 mmol/L, *p* = 0.036), while the TyG index was higher (8.74 vs. 8.45, *p* = 0.016). In addition, the OSA group had a higher proportion of smoking (35.00% vs. 16.13%, *p* = 0.039), drinking (25.62% vs. 9.68%, *p* = 0.054, borderline significant) and hypertension (45.00% vs. 25.81%, *p* = 0.047), but no significant difference in the prevalence of cardiovascular disease and stroke. At the same time, there was no significant difference in the use of lipid-lowering drugs between the two groups (*p* > 0.05).

**Table 1:** Characteristics of the study population.

Variables	Total	No OSA *n* = 31 (16.23%)	OSA *n* = 160 (83.77%)	*p*-value
Age (y)	48.94 (8.91)	48.97 (10.25)	48.93 (8.67)	0.983
Body mass index (kg/m^2^)	25.23 (3.05)	24.27 (3.56)	25.41 (2.92)	0.056
AHI (/h)	17.50 (7.15–32.65)	2.70 (1.70–3.35)	22.15 (11.25–37.47)	**<0.001**
ODI (/h)	18.80 (9.40–32.20)	4.30 (2.30–7.40)	22.55 (12.20–35.23)	**<0.001**
Mean SPO_2_ (%)	94.24 (2.00)	95.26 (1.46)	94.04 (2.04)	**0.002**
LSPO_2_ (%)	78.69 (10.98)	87.91 (3.91)	76.90 (11.02)	**<0.001**
T90 (%)	3.42 (0.70–8.75)	0.09 (0.00–0.68)	4.78 (1.31–10.22)	**<0.001**
Fasting plasma glucose (mmol/L)	4.79 (0.69)	4.65 (0.59)	4.82 (0.70)	0.187
Total cholesterol (mmol/L)	4.53 (0.92)	4.49 (0.98)	4.54 (0.92)	0.762
Triglycerides (mmol/L)	1.51 (1.06–2.24)	1.23 (0.88–1.89)	1.56 (1.09–2.32)	0.055
HDL-C (mmol/L)	1.01 (0.30)	1.12 (0.44)	0.99 (0.26)	**0.036**
Non-HDL-C (mmol/L)	3.52 (0.85)	3.37 (0.91)	3.55 (0.84)	0.287
LDL-C (mmol/L)	2.61 (0.71)	2.67 (0.91)	2.60 (0.67)	0.598
TyG index	8.69 (0.60)	8.45 (0.54)	8.74 (0.60)	**0.016**
Sex, n (%)				0.084
Male	130 (68.06%)	17 (54.84%)	113 (70.62%)	
Female	61 (31.94%)	14 (45.16%)	47 (29.38%)	
Smoking history, n (%)	61 (31.94%)	5 (16.13%)	56 (35.00%)	**0.039**
Alcohol drinking, n (%)	44 (23.04%)	3 (9.68%)	41 (25.62%)	0.054
Hypertension, n (%)	80 (41.88%)	8 (25.81%)	72 (45.00%)	**0.047**
Cardiovascular diseases, n (%)	34 (17.80%)	2 (6.45%)	32 (20.00%)	0.071
Stroke, n (%)	12 (6.28%)	0 (0.00%)	12 (7.50%)	0.115
Use lipid-lowering drugs, n (%)	41 (21.47%)	3 (9.68%)	38 (23.75%)	0.081

AHI, apnea-hypopnea index; HDL-C, high-density lipoprotein cholesterol; LDL-C, low-density lipoprotein cholesterol; TyG, triglyceride-glucose. Categorical variables were expressed as numbers (percentage, %), and continuous variables were described as means (standard deviation, SD) or medians (interquartile range, IQR), as appropriate. One-way analysis of variance (normal distribution), Kruskal-Wallis test (skewed distribution), and chi-square test (categorical variables) were used to compare differences between groups. Significant results are in bold.

To further analyze the sex differences, the baseline characteristics of male participants (*n* = 130, 68.06%) and female participants (*n* = 61, 31.94%) were compared in Table 2. Compared with female participants, male participants were younger (47.97 vs. 51.00 years, *p* = 0.028), but had significantly higher OSA severity (AHI 19.90 vs. 10.80 /hour, *p* < 0.001; ODI 21.10 vs. 15.30 /hour, *p* = 0.025) and more severe hypoxemia (LSPO₂ 77.14% vs. 81.98%, *p* = 0.004; T90 4.35% vs. 1.99%, *p* = 0.012). More importantly, males showed more obvious metabolic disorders (triglycerides 1.58 vs. 1.20 mmol/L, *p* = 0.032; HDL-C 0.95 vs. 1.15 mmol/L, *p* < 0.001; TyG index 8.77 vs. 8.53, *p* = 0.011). In addition, males had significantly higher rates of smoking (45.38% vs. 3.28%, *p* < 0.001), drinking (33.08% vs. 1.64%, *p* < 0.001) and hypertension (47.69% vs. 29.51%, *p* = 0.018) than females, but there was no significant difference in the prevalence of cardiovascular disease and stroke between the two groups (all *p* > 0.05). There was also no significant difference in the use of lipid-lowering drugs and the prevalence of OSA between the two groups (*p* > 0.05).

**Table 2:** Characteristics of the female and male participants.

Variables	Male *n* = 130 (68.06%)	Female *n* = 61 (31.94%)	*p*-value
Age (y)	47.97 (9.21)	51.00 (7.92)	**0.028**
Body mass index (kg/m^2^)	25.43 (3.02)	24.79 (3.10)	0.173
AHI (/h)	19.90 (9.33–39.40)	10.80 (5.10–25.30)	**<0.001**
ODI (/h)	21.10 (9.83–33.48)	15.30 (8.70–30.60)	**0.025**
Mean SPO_2_ (%)	94.05 (2.15)	94.63 (1.58)	0.062
LSPO_2_ (%)	77.14 (12.10)	81.98 (7.13)	**0.004**
T90 (%)	4.35 (0.82–10.12)	1.99 (0.33–7.63)	**0.012**
Fasting plasma glucose (mmol/L)	4.82 (0.68)	4.74 (0.71)	0.450
Total cholesterol (mmol/L)	4.47 (0.89)	4.66 (0.99)	0.199
Triglycerides (mmol/L)	1.58 (1.11–2.33)	1.20 (1.01–1.80)	**0.032**
HDL-C(mmol/L)	0.95 (0.28)	1.15 (0.31)	**<0.001**
Non-HDL-C (mmol/L)	3.52 (0.83)	3.51 (0.91)	0.922
LDL-C (mmol/L)	2.59 (0.72)	2.67 (0.70)	0.453
TyG index	8.77 (0.60)	8.53 (0.57)	**0.011**
Smoking history, n (%)	59 (45.38%)	2 (3.28%)	**<0.001**
Alcohol drinking, n (%)	43 (33.08%)	1 (1.64%)	**<0.001**
Hypertension, n (%)	62 (47.69%)	18 (29.51%)	**0.018**
Cardiovascular diseases, n (%)	25 (19.23%)	9 (14.75%)	0.451
Stroke, n (%)	8 (6.15%)	4 (6.56%)	0.915
Use lipid-lowering drugs, n (%)	30 (23.08%)	11 (18.03%)	0.429
OSA, n (%)	113 (86.92%)	47 (77.05%)	0.084

AHI, apnea-hypopnea index; HDL-C, high-density lipoprotein cholesterol; LDL-C, low-density lipoprotein cholesterol; TyG, triglyceride-glucose. Categorical variables were expressed as numbers (percentage, %), and continuous variables were described as means (standard deviation, SD) or medians (interquartile range, IQR), as appropriate. One-way analysis of variance (normal distribution), Kruskal-Wallis test (skewed distribution), and chi-square test (categorical variables) were used to compare differences between groups. Significant results are in bold.

**Figure 1:**
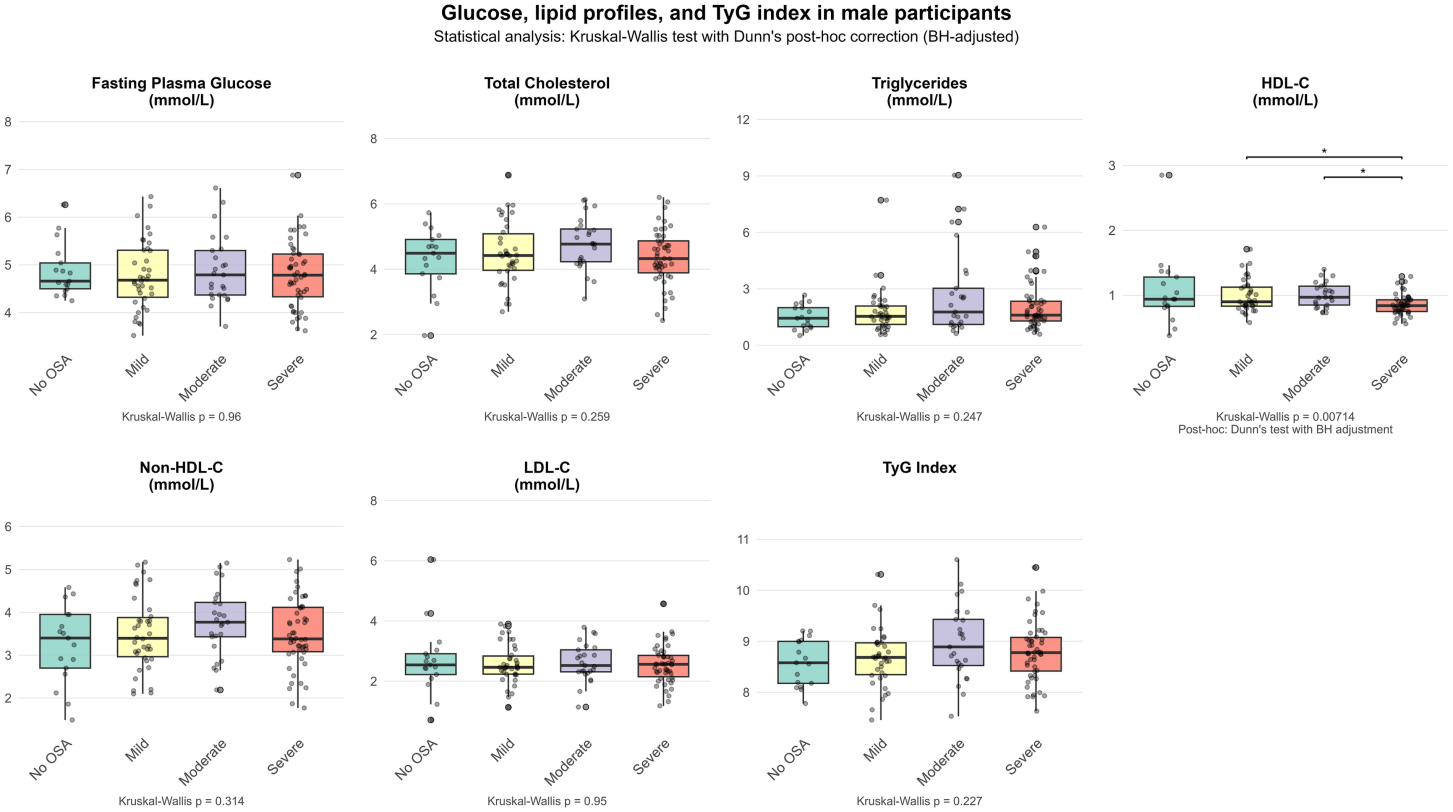
Glucose, lipid profiles, and TyG index in male participants.

**Figure 2:**
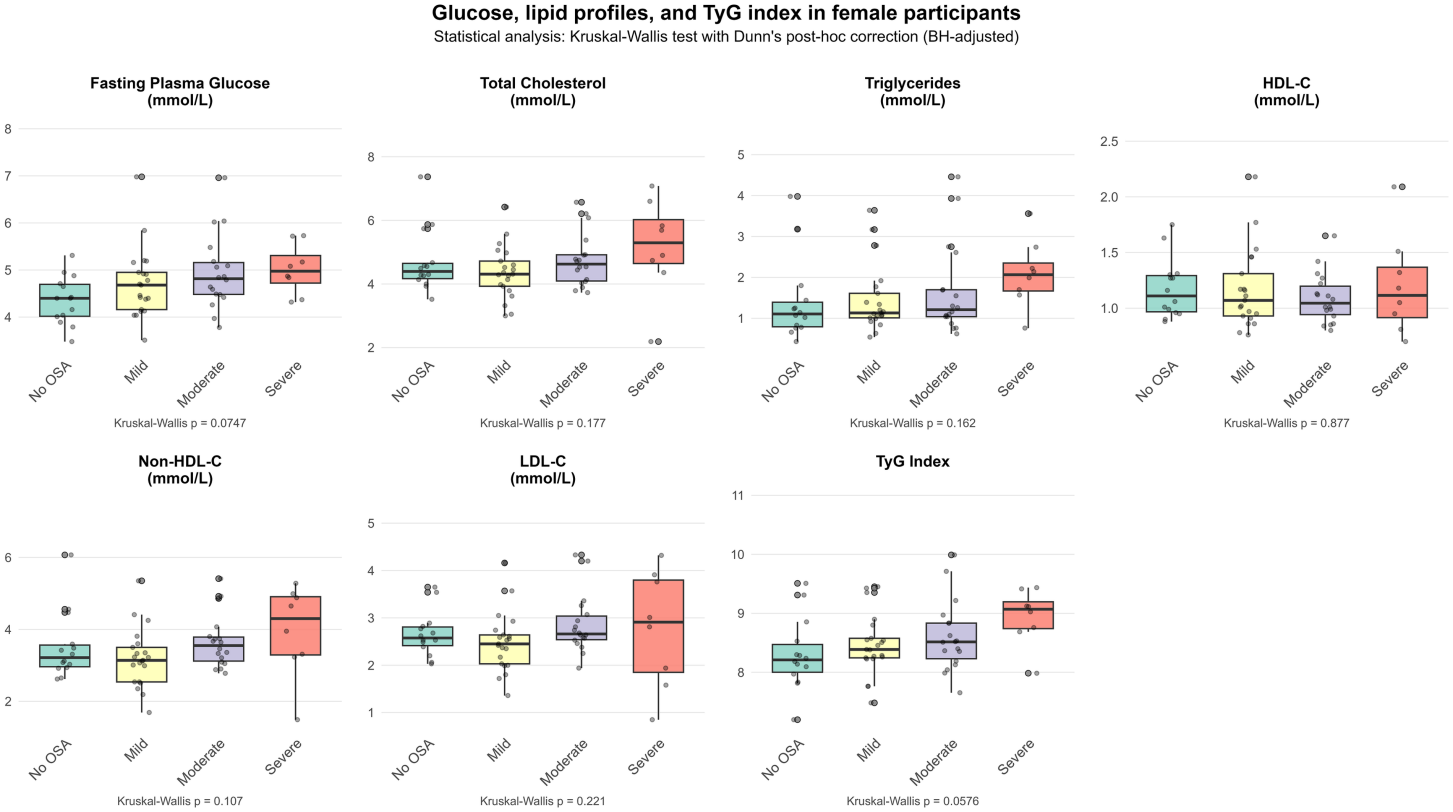
Glucose, lipid profiles, and TyG index in female participants.

Figures 1, 2 show the differential distribution of FPG, lipid profiles, and TyG index in different OSA severity groups in males and females, respectively, in the form of box plots.

Table 3 shows the results of Spearman correlation analysis between AHI and FPG, lipid profiles, and TyG index, which are presented by sex (male, female) and total population. In males, only HDL-C was significantly negatively correlated with AHI (*r* = −0.194, *p* = 0.027), while females showed that FPG (*r* = 0.373, *p* = 0.003), non-HDL-C (*r* = 0.280, *p* = 0.029) and TyG index (*r* = 0.337, *p* = 0.008) were significantly positively correlated with AHI. In the total population, triglycerides (*r* = 0.212, *p* = 0.003) and TyG index (*r* = 0.230, *p* = 0.001) were significantly positively correlated with AHI, while HDL-C was significantly negatively correlated with AHI (*r* = −0.263, *p* < 0.001).

**Table 3:** Spearman correlation analysis of AHI with glucose, lipid profile, and TyG index.

Variables		Male	Female	Total
Fasting plasma glucose (mmol/L)	*r*	−0.003	0.373	0.121
	*p*	0.971	0.003**	0.094
Total cholesterol (mmol/L)	*r*	−0.049	0.230	0.013
	*p*	0.582	0.075	0.853
Triglycerides (mmol/L)	*r*	0.132	0.235	0.212
	*p*	0.133	0.068	0.003**
HDL-C (mmol/L)	*r*	−0.194	−0.085	−0.263
	*p*	0.027*	0.513	<0.001***
Non-HDL-C (mmol/L)	*r*	0.031	0.280	0.114
	*p*	0.730	0.029*	0.117
LDL-C (mmol/L)	*r*	−0.017	0.203	0.016
	*p*	0.846	0.116	0.823
TyG index	*r*	0.124	0.337	0.230
	*p*	0.161	0.008**	0.001**

Spearman correlation analysis was used to determine the relationship between AHI and glycolipid parameters. *p < 0.05; **p < 0.01; ***p < 0.001. AHI, apnea-hypopnea index; HDL-C, high-density lipoprotein cholesterol; LDL-C, low-density lipoprotein cholesterol; TyG, triglyceride-glucose. Significant results are in bold.

Table 4 presents the results of multivariable regression analyses examining the associations between OSA and glucose metabolism in males, females, and the overall population. Compared with non-OSA female participants, female AHI ≥ 5 group (*β* = 0.55, 95% CI 0.13 to 0.98, *p* = 0.0143 and AHI ≥ 15 group (*β* = 0.60, 95% CI 0.13 to 1.07, *p* = 0.0148) were significantly associated with FPG, and the group AHI ≥ 30 was also close to significant difference (*p* = 0.0504). In contrast, no statistically significant independent associations were found between any OSA severity group and FPG levels in male participants and overall population analysis (all *p* > 0.05).

**Table 4:** The association between OSA severity and fasting plasma glucose, stratified by sex.

Variable	Male	Female	Total
	β (95%CI) *p*-value	*β* (95%CI) *p*-value	β (95%CI) *p*-value
Group 1			
*AHI<5*	Reference	Reference	Reference
*AHI≥5*	−0.11 (−0.48, 0.25) 0.5399	**0.55 (0.13, 0.98) 0.0143**	0.17 (−0.11, 0.44) 0.2311
Group 2			
*AHI<5*	Reference	Reference	Reference
*5 ≤ AHI<15*	−0.10 (−0.50, 0.29) 0.6129	0.48 (−0.02, 0.99) 0.0645	0.13 (−0.17, 0.44) 0.3945
*AHI≥15*	−0.13 (−0.52, 0.27) 0.5318	**0.60 (0.13, 1.07) 0.0148**	0.19 (−0.10, 0.49) 0.1924
Group 3			
*AHI<5*	Reference	Reference	Reference
*5 ≤ AHI<15*	−0.10 (−0.50, 0.30) 0.6132	0.49 (−0.02, 0.99) 0.0655	0.13 (−0.17, 0.43) 0.4062
*15 ≤ AHI<30*	−0.09 (−0.55, 0.36) 0.6956	**0.57 (0.06, 1.08) 0.0320**	0.24 (−0.09, 0.57) 0.1508
*AHI≥30*	−0.14 (−0.55, 0.27) 0.4981	0.68 (0.02, 1.34) 0.0504	0.15 (−0.17, 0.47) 0.3633

Significant results are in bold. Abbreviation: β, effect size; 95% Cl 95%, confidence interval; OSA, obstructive sleep apnea; AHI, apnea-hypopnea index. All subgroups were adjusted for age, sex, body mass index, smoking history, alcohol drinking, hypertension, cardiovascular diseases, stroke, and use lipid-lowering drugs, except the stratification factor itself.

Table 5 further shows the relationship between OSA and triglyceride levels. Among males, only the 15 ≤ AHI < 30 group was significantly positively associated with triglyceride levels (*β* = 1.00, 95% CI 0.05 to 1.95, *p* = 0.0403), and no other groups showed significant associations (all *p* > 0.05). In females, there was no significant association between each OSA group and triglyceride levels (all *p* > 0.05). In the overall population, the 15 ≤ AHI < 30 group was independently positively associated with triglyceride levels (*β* = 0.64, 95% CI 0.03 to 1.24, *p* = 0.0402). In addition, the association between each OSA severity group and HDL-C level also showed sex differences (Table 6): among males, the AHI ≥ 15 group (*β* = −0.17, 95% CI −0.33 to −0.01, *p* = 0.0363) and the AHI ≥ 30 group (*β* = −0.22, 95% CI −0.38 to −0.06, *p* = 0.0100) were both significantly negatively associated with HDL-C, while among females, no significant association was found between each OSA severity group and HDL-C level (all *p* > 0.05).

**Table 5:** The association between OSA severity and triglyceride, stratified by sex.

Variable	Male	Female	Total
	β (95%CI) *p*-value	β (95%CI) *p*-value	β (95%CI) *p*-value
Group 1			
*AHI<5*	Reference	Reference	Reference
*AHI≥5*	0.38 (−0.39, 1.15) 0.3363	0.36 (−0.20, 0.91) 0.2135	0.32 (−0.19, 0.83) 0.2172
Group 2			
*AHI<5*	Reference	Reference	Reference
*5 ≤ AHI<15*	0.24 (−0.60, 1.07) 0.5804	0.30 (−0.36, 0.95) 0.3782	0.18 (−0.38, 0.74) 0.5334
*AHI≥15*	0.51 (−0.31, 1.34) 0.2272	0.40 (−0.21, 1.01) 0.2034	0.43 (−0.11, 0.97) 0.1208
Group 3			
*AHI<5*	Reference	Reference	Reference
*5 ≤ AHI<15*	0.23 (−0.59, 1.06) 0.5831	0.30 (−0.35, 0.96) 0.3700	0.17 (−0.40, 0.73) 0.5630
*15 ≤ AHI<30*	**1.00 (0.05, 1.95) 0.0403**	0.33 (−0.33, 0.99) 0.3310	**0.64 (0.03, 1.24) 0.0402**
*AHI≥30*	0.28 (−0.57, 1.13) 0.5206	0.57 (−0.29, 1.43) 0.1970	0.23 (−0.37, 0.83) 0.4475

Significant results are in bold. β, effect size; 95% Cl 95%, confidence interval; OSA, obstructive sleep apnea; AHI, apnea-hypopnea index. All subgroups were adjusted for age, sex, body mass index, smoking history, alcohol drinking, hypertension, cardiovascular diseases, stroke, and use lipid-lowering drugs, except the stratification factor itself.

**Table 6:** The association between OSA severity and HDL-C, stratified by sex.

Variable	Male	Female	Total
	β (95%CI) *p*-value	β (95%CI) *p*-value	β (95%CI) *p*-value
Group 1			
*AHI<5*	Reference	Reference	Reference
*AHI≥5*	−0.13 (−0.28, 0.02) 0.0956	−0.00 (−0.20, 0.20) 0.9849	−0.07 (−0.19, 0.04) 0.2040
Group 2			
*AHI<5*	Reference	Reference	Reference
*5 ≤ AHI<15*	−0.08 (−0.24, 0.08) 0.3294	0.03 (−0.21, 0.26) 0.8283	−0.04 (−0.17, 0.09) 0.5393
*AHI≥15*	**−0.17 (−0.33, −0.01) 0.0363**	−0.02 (−0.24, 0.19) 0.8444	−0.10 (−0.22, 0.02) 0.1052
Group 3			
*AHI<5*	Reference	Reference	Reference
*5 ≤ AHI<15*	−0.08 (−0.24, 0.08) 0.3176	0.03 (−0.20, 0.26) 0.7802	−0.04 (−0.17, 0.08) 0.5157
*15 ≤ AHI<30*	−0.08 (−0.26, 0.11) 0.4174	−0.09 (−0.32, 0.14) 0.4631	−0.07 (−0.20, 0.07) 0.3460
*AHI≥30*	**−0.22 (−0.38, −0.06) 0.0100**	0.14 (−0.16, 0.44) 0.3687	−0.14 (−0.27, −0.00) 0.0502

Significant results are in bold. β, effect size; 95% Cl 95%, confidence interval; OSA, obstructive sleep apnea; AHI, apnea-hypopnea index; HDL-C, high-density lipoprotein cholesterol. All subgroups were adjusted for age, sex, body mass index, smoking history, alcohol drinking, hypertension, cardiovascular diseases, stroke, and use lipid-lowering drugs, except the stratification factor itself.

Table 7 shows the association between OSA severity and TyG index. In female participants, AHI ≥ 5 group showed a significant association in TyG index (*β* = 0.37, 95% CI 0.08 to 0.66, *p* = 0.0164), and a dose–response relationship was observed with OSA severity (AHI ≥ 15 group: *β* = 0.39, 95% CI 0.07 to 0.71, *p* = 0.0197; AHI ≥ 30 group: *β* = 0.53, 95% CI 0.08 to 0.98, *p* = 0.0258). However, there was no significant association between OSA groups and TyG index in male participants (all *p* > 0.05).

**Table 7:** The association between OSA severity and TyG index, stratified by sex.

Variable	Male	Female	Total
	β (95%CI) *p*-value	β (95%CI) *p*-value	*β* (95%CI) *p*-value
Group 1			
*AHI<5*	Reference	Reference	Reference
*AHI≥5*	0.08 (−0.24, 0.40) 0.6186	**0.37 (0.08, 0.66) 0.0164**	0.17 (−0.06, 0.39) 0.1456
Group 2			
*AHI<5*	Reference	Reference	Reference
*5 ≤ AHI<15*	0.05 (−0.30, 0.39) 0.7944	0.34 (−0.01, 0.68) 0.0606	0.12 (−0.13, 0.37) 0.3387
*AHI≥15*	0.11 (−0.23, 0.45) 0.5176	**0.39 (0.07, 0.71) 0.0197**	0.20 (−0.04, 0.44) 0.0993
Group 3			
*AHI<5*	Reference	Reference	Reference
*5 ≤ AHI<15*	0.04 (−0.30, 0.39) 0.7991	0.34 (−0.00, 0.69) 0.0575	0.12 (−0.13, 0.37) 0.3521
*15 ≤ AHI<30*	0.24 (−0.16, 0.63) 0.2425	0.34 (−0.01, 0.69) 0.0600	0.25 (−0.02, 0.52) 0.0677
*AHI≥30*	0.05 (−0.30, 0.41) 0.7650	**0.53 (0.08, 0.98) 0.0258**	0.15 (−0.11, 0.42) 0.2536

Significant results are in bold. β, effect size; 95% Cl 95%, confidence interval; OSA, obstructive sleep apnea; AHI, apnea-hypopnea index; TyG, triglyceride-glucose. All subgroups were adjusted for age, sex, body mass index, smoking history, alcohol drinking, hypertension, cardiovascular diseases, stroke, and use lipid-lowering drugs, except the stratification factor itself.

In the sex-stratified and overall population analyses, OSA severity did not show significant associations with total cholesterol, non-HDL-C, and LDL-C levels (all *p* > 0.05; Supplementary Tables 1–3).

## 4 Discussion

To our knowledge, this is the first study to investigate the glucose and lipid metabolism in non-diabetic, non-obese OSA participants in the Chinese population. We found that after adjusting for potential confounding factors including age, BMI, smoking history, alcohol drinking, comorbidities, and use of lipid-lowering drugs, non-diabetic, non-obese OSA participants had significant disorders in glucose and lipid metabolism, and showed obvious sex differences. It is noteworthy that the severity of OSA in females was significantly positively associated with FPG level and TyG index. In contrast, the severity of OSA in males was negatively associated with HDL-C level and positively associated with triglyceride level.

Dyslipidemia in the Chinese population has shown a unique upward trend. Over the past 40 years, non-HDL-C levels have increased significantly, and the global ranking has jumped from 153rd to 99th (22). The prevalence of dyslipidemia has increased sharply from 18.6% in 2002 to 33.8% in 2014–2019. During the same period, the obesity and diabetes prevalence rates have also shown a rapid upward trend: the adult obesity rate increased from 4.2% in 1993 to 15.7% in 2015, and the diabetes prevalence rate climbed from less than 1% in 1980 to 12.4% in 2018 (23–25).

Dyslipidemia is common in OSA and is often attributed to obesity and/or diabetes, which is also a common comorbidity of OSA. Lipid metabolism disorders are the basis for the development of atherosclerosis and are closely related to cardiovascular disease-related morbidity and mortality (26). Systematic reviews and meta-analyses have shown that OSA patients have higher total cholesterol, LDL, and triglycerides, lower HDL, and a higher risk of dyslipidemia (27). Studies in animal models and OSA patients have revealed that intermittent hypoxia and sleep fragmentation in OSA are potential mechanisms linking it to metabolic dysfunction (28). Of note, in most studies, the most prominent lipid abnormality in OSA patients is hypertriglyceridemia, which is well-known and is often attributed to the high prevalence of obesity in the OSA population, as high triglycerides are directly related to obesity and carbohydrate metabolism. Interestingly, our study population also had similar findings after excluding patients with obesity and diabetes, that is, OSA patients had higher triglyceride levels compared with non-OSA patients (although *p* = 0.055, the sample size was small and the result was borderline significant).

However, the relationship between OSA and metabolic disorders may have racial differences (29). A multiethnic study revealed that Chinese participants showed different associations with fasting glucose metabolism from other ethnic groups. The study found that although moderate to severe OSA was strongly associated with abnormal fasting glucose in African Americans and White people, no significant association was observed in the Chinese population (race interaction *p* = 0.06) (29). At the same time, the study did not find evidence of an interaction between obesity and OSA (*p* = 0.68). After accounting for obesity, the interaction between race and OSA still existed (*p* = 0.03) (30). However, another Korean cohort study revealed significant obesity differences in the metabolic effects of OSA: in non-obese individuals, OSA was significantly associated with impaired fasting glucose, impaired glucose tolerance and diabetes risk, and this association remained after accounting for visceral fat. In contrast, in obese participants, OSA was not significantly associated with any abnormal glucose tolerance category (31).

Therefore, despite the inconsistent conclusions of existing studies, obesity may still be an important modifier of the association between OSA and metabolic disorders. Considering that the prevalence of obesity in the Chinese population is lower than that in Western populations and that this association may have racial differences, this further emphasizes the necessity and clinical significance of studying OSA-related metabolic disorders in non-obese Chinese populations. To our knowledge, only two studies have specifically investigated glucose and lipid metabolism in nondiabetic, nonobese OSA populations. Basoglu et al. pointed out that non-obese, non-diabetic OSA patients had significant dyslipidemia (elevated total cholesterol, LDL-C, non-HDL-C, and triglycerides), and that the correlation between lipid profiles and OSA severity was stronger in females than in males (32). Another study confirmed that OSA was independently associated with elevated TyG index in non-diabetic, non-obese people (33). In our study, we observed that the severity of OSA in females was significantly positively associated with FPG levels and TyG index; whereas the severity of OSA in males was negatively associated with HDL-C levels and positively associated with triglyceride levels. In addition, we did not observe an independent association between OSA and TyG index in the overall population.

The differences in research results may be caused by multiple factors. First, it is necessary to consider the impact of ethnic and regional factors on serum lipid profiles, because studies have shown that serum lipid levels vary among people of different ethnicities and regions (34–36). Unlike Western countries (such as the United States), where dyslipidemia is mainly manifested as increased total cholesterol and LDL-C, dyslipidemia in the Chinese population is more often manifested as increased triglycerides and decreased HDL-C (37). Although in our current analysis, geographic differences alone could not explain the inconsistent conclusions of studies on the effects of OSA on lipid. Second, the sample size limitation of this study resulted in the failure to exclude patients who were receiving lipid-lowering treatment. Although the use of lipid-lowering drugs was corrected in the statistical analysis, its potential impact cannot be completely eliminated, especially considering that statins may improve serum lipid metabolism by restoring complement-mediated endothelial protection and inhibiting downstream proinflammatory responses (38). Third, differences in baseline characteristics of participants between studies, including age distribution, sex ratio, and severity of OSA, may have affected the study results.

Another interesting finding of this study is that although the severity of OSA in female participants was milder than that in male participants, the correlation between their metabolic indicators and AHI was more significant: FPG, non-HDL-C and TyG index are all significantly positively correlated with AHI. In contrast, male participants only showed a significant negative correlation between HDL-C and AHI. This finding is very similar to the results observed in the study of Basoglu et al. (32). These differences suggest that OSA may affect lipid metabolism through sex-specific pathophysiological mechanisms, with men being more prone to traditional dyslipidemia, while women are more prominent in glucose metabolism disorders and insulin resistance. Sex hormones are a factor that may differentially regulate glucose and lipid metabolism disorder in men and women (39). In women, elevated total estradiol levels are associated with an increased risk of diabetes, which may explain the more frequent presence of dysregulated glucose metabolism in women with OSA (40). Similarly, there are differences between men and women in terms of lipids and lipoproteins (41). Adult and middle-aged men are more likely to have elevated LDL-C levels and reduced HDL-C levels (42).

These hormonal influences may interact with pathophysiological changes caused by OSA, resulting in different metabolic phenotypes between the sexes. Despite lower overall OSA severity in females, metabolic parameters were more strongly associated with AHI, suggesting that the metabolic consequences of sleep-disordered breathing may be more sensitive in females.

It is worth noting that our study did not find a significant association between OSA and non-HDL-C levels in the overall population, which is different from the results of previous studies. Existing evidence shows that atherosclerotic dyslipidemia is quite common in OSA patients, especially non-HDL-C (but not LDL-C), which is significantly related with OSA severity and hypoxia parameters (43). The negative results of our study may be due to the exclusion criteria for patients with diabetes and obesity, which to some extent changed the baseline characteristics of the study population. On the other hand, racial and regional differences are also a factor. Therefore, when interpreting the results of OSA-related dyslipidemia studies, the differences in study population and race and region should be fully considered.

### 4.1 Strengths and limitations

This study has several strengths. First, we evaluated OSA using the HSAT device. Unlike subjective questionnaires, this device-based approach is able to measure apnea-related indicators. Second, we excluded participants with T2DM and obesity. Third, our regression analysis included multiple potential confounders, including demographic factors, smoking and drinking, comorbidities, and the use of lipid-lowering drugs, which improved the robustness of the study results.

However, there are also some limitations. First, the exclusion of patients with diabetes and obesity resulted in a reduced sample size, which may have reduced the power of the statistical analysis. Second, although the HSAT device used has been validated for sleep monitoring, its accuracy in sleep staging, arousal detection, and sleep structure analysis is still inferior to that of polysomnography. Third, the retrospective design limits the collection of confounding factors, including variables such as diet structure, exercise habits, sex hormone levels, and menopausal status, which are particularly important for analyzing sex differences. Fourth, although the TyG index has been proven to be a reliable alternative to HOMA-IR, the lack of directly measured HOMA-IR levels is still a limitation. Similarly, due to the limitation of retrospective data, the lack of HbA1c test results makes it impossible to evaluate the relationship between OSA and long-term fasting glucose levels. Fifth, although this study found that OSA severity was statistically associated with glucose and lipid metabolism, it should be noted that statistical significance is not equivalent to clinical relevance. In addition, the relatively small sample size of the female subgroup may affect the credibility of sex-specific analysis, so the interpretation of these statistical results should be cautious. Finally, the nature of the cross-sectional study determines that the causal relationship between OSA and metabolic disorders cannot be established. As a single-center study in a tertiary hospital, the extrapolation of its conclusions may be limited, especially in primary care and community populations. Future longitudinal studies with larger samples and multiple centers are needed to further validate the findings of our study.

## 5 Conclusion

In conclusion, this study showed that there were sex differences in metabolic disorders in non-diabetic, non-obese OSA participants. Females were mainly characterized by increased FPG and TyG index, while males were mainly characterized by dyslipidemia with increased triglycerides and decreased HDL-C. These findings suggest that sex differences may need to be considered when assessing OSA-related metabolic risks. However, this study was designed as a cross-sectional study and therefore can only demonstrate associations between variables, but not causality. Likewise, as this study was conducted in a tertiary hospital, the generalizability of these results to community populations may be limited. Further multicenter cohort studies involving different populations are needed to validate these observations.

## Data Availability

The raw data supporting the conclusions of this article will be made available by the authors, without undue reservation.

## Footnotes

^1^ http://www.R-project.org

^2^ http://www.empowerstats.com
