# Supplementary material for: Glucose and lipid metabolism in non-diabetic, non-obese patients with obstructive sleep apnea: sex differences
Source: Front Nutr. 2025 Aug 20;12:1619371. doi: 10.3389/fnut.2025.1619371 (PMC12404915; doi:10.3389/fnut.2025.1619371)
Supplement: Supplementary file 1 [file Table_1.docx]

**Supplementary Table 1. The association between OSA severity and total cholesterol, stratified by sex.**

| **Variable** | Male | Female | Total |
| --- | --- | --- | --- |
|  | β (95%CI) P-value | β (95%CI) P-value | β (95%CI) P-value |
| **Group 1** | | | |
| *AHI<5* | Reference | Reference | Reference |
| *AHI≥5* | 0.00 (-0.46, 0.47) 0.9886 | 0.14 (-0.43, 0.71) 0.6253 | 0.00 (-0.35, 0.35) 0.9875 |
| **Group 2** | | | |
| *AHI<5* | Reference | Reference | Reference |
| *5≤AHI<15* | 0.10 (-0.41, 0.60) 0.7108 | -0.07 (-0.73, 0.59) 0.8318 | -0.02 (-0.41, 0.37) 0.9177 |
| *AHI≥15* | -0.08 (-0.58, 0.42) 0.7519 | 0.30 (-0.32, 0.91) 0.3498 | 0.02 (-0.35, 0.39) 0.9136 |
| **Group 3** | | | |
| *AHI<5* | Reference | Reference | Reference |
| *5≤AHI<15* | 0.09 (-0.41, 0.59) 0.7155 | -0.06 (-0.71, 0.60) 0.8670 | -0.03 (-0.42, 0.36) 0.8870 |
| *15≤AHI<30* | 0.17 (-0.41, 0.74) 0.5709 | 0.15 (-0.51, 0.82) 0.6478 | 0.14 (-0.28, 0.55) 0.5155 |
| *AHI≥30* | -0.20 (-0.71, 0.31) 0.4508 | 0.65 (-0.21, 1.51) 0.1474 | -0.09 (-0.51, 0.32) 0.6589 |

Significant results are in bold.

Abbreviation: β, effect size; 95% Cl 95%, confidence interval; OSA, obstructive sleep apnea; AHI, apnea-hypopnea index.

All subgroups were adjusted for age, sex, body mass index, smoking history, alcohol drinking, hypertension, cardiovascular diseases, stroke, and use lipid-lowering drugs, except the stratification factor itself.

**Supplementary Table 2. The association between OSA severity and non-HDL-C, stratified by sex.**

| **Variable** | Male | Female | Total |
| --- | --- | --- | --- |
|  | β (95%CI) P-value | β (95%CI) P-value | β (95%CI) P-value |
| **Group 1** | | | |
| *AHI<5* | Reference | Reference | Reference |
| *AHI≥5* | 0.13 (-0.30, 0.56) 0.5453 | 0.14 (-0.37, 0.66) 0.5831 | 0.08 (-0.24, 0.40) 0.6359 |
| **Group 2** | | | |
| *AHI<5* | Reference | Reference | Reference |
| *5≤AHI<15* | 0.18 (-0.29, 0.64) 0.4590 | -0.10 (-0.69, 0.49) 0.7467 | 0.02 (-0.34, 0.38) 0.9150 |
| *AHI≥15* | 0.09 (-0.37, 0.55) 0.6925 | 0.32 (-0.23, 0.87) 0.2621 | 0.12 (-0.22, 0.46) 0.4842 |
| **Group 3** | | | |
| *AHI<5* | Reference | Reference | Reference |
| *5≤AHI<15* | 0.18 (-0.29, 0.64) 0.4623 | -0.09 (-0.68, 0.50) 0.7690 | 0.01 (-0.34, 0.37) 0.9384 |
| *15≤AHI<30* | 0.24 (-0.29, 0.77) 0.3754 | 0.24 (-0.35, 0.84) 0.4298 | 0.20 (-0.18, 0.59) 0.2960 |
| *AHI≥30* | 0.02 (-0.46, 0.50) 0.9280 | 0.51 (-0.27, 1.28) 0.2052 | 0.04 (-0.34, 0.42) 0.8247 |

Significant results are in bold.

Abbreviation: β, effect size; 95% Cl 95%, confidence interval; OSA, obstructive sleep apnea; AHI, apnea-hypopnea index; non-HDL-C, non-high-density lipoprotein cholesterol.

All subgroups were adjusted for age, sex, body mass index, smoking history, alcohol drinking, hypertension, cardiovascular diseases, stroke, and use lipid-lowering drugs, except the stratification factor itself.

**Supplementary Table 3. The association between OSA severity and LDL-C, stratified by sex.**

| **Variable** | Male | Female | Total |
| --- | --- | --- | --- |
|  | β (95%CI) P-value | β (95%CI) P-value | β (95%CI) P-value |
| **Group 1** | | | |
| *AHI<5* | Reference | Reference | Reference |
| *AHI≥5* | -0.25 (-0.63, 0.12) 0.1906 | 0.09 (-0.33, 0.51) 0.6764 | -0.13 (-0.40, 0.14) 0.3477 |
| **Group 2** | | | |
| *AHI<5* | Reference | Reference | Reference |
| *5≤AHI<15* | -0.19 (-0.59, 0.22) 0.3691 | -0.11 (-0.59, 0.38) 0.6651 | -0.15 (-0.45, 0.15) 0.3336 |
| *AHI≥15* | -0.31 (-0.71, 0.09) 0.1312 | 0.23 (-0.22, 0.68) 0.3190 | -0.12 (-0.41, 0.17) 0.4343 |
| **Group 3** | | | |
| *AHI<5* | Reference | Reference | Reference |
| *5≤AHI<15* | -0.19 (-0.60, 0.22) 0.3705 | -0.11 (-0.60, 0.38) 0.6541 | -0.15 (-0.46, 0.15) 0.3192 |
| *15≤AHI<30* | -0.29 (-0.75, 0.18) 0.2324 | 0.28 (-0.21, 0.77) 0.2749 | -0.05 (-0.37, 0.28) 0.7855 |
| *AHI≥30* | -0.32 (-0.74, 0.10) 0.1327 | 0.12 (-0.51, 0.76) 0.7058 | -0.19 (-0.51, 0.14) 0.2622 |

Significant results are in bold.

Abbreviation: β, effect size; 95% Cl 95%, confidence interval; OSA, obstructive sleep apnea; AHI, apnea-hypopnea index; LDL-C, low-density lipoprotein cholesterol.

All subgroups were adjusted for age, sex, body mass index, smoking history, alcohol drinking, hypertension, cardiovascular diseases, stroke, and use lipid-lowering drugs, except the stratification factor itself.
